# Tuning the thermostat. Beyond hot and cold in the medulloblastoma tumor microenvironment

**DOI:** 10.1016/j.ebiom.2025.106101

**Published:** 2025-12-27

**Authors:** Allison M. Martin

**Affiliations:** Albert Einstein College of Medicine, Pediatrics, Microbiology & Immunology, 1300 Morris Park Avenue, Van Etten 6A04B, Bronx, NY, 10461, USA

The “cold” tumor microenvironment in medulloblastoma has been a point of fascination and frustration for the neuro immuno oncology community since it first became clear that this tumor had a significantly lower immune infiltrate than other pediatric brain tumors.^1^^,^^2^ In a world where immunotherapy has unlocked so many doors for other cancers, it has been a struggle to bring these benefits to patients with medulloblastoma, to turn up the heat, so to speak. While it is intuitive that more infiltrating immune cells should predict better responses to immunotherapy, small sample sizes have made this hypothesis difficult to confirm in medulloblastoma. Chen and colleagues have overcome this barrier by assembling a large clinically annotated cohort of medulloblastoma patients and analyzing them at the protein level.^3^ While other studies have primarily focused on the total number of infiltrating immune cells, this study is unique in that it evaluated the density of immune cells within tumor cores and how not just their numbers but their proximity to other cells influences outcomes. Predictably, high density of proliferating CD3 and CD4 T cells was associated with increased survival across all subgroups while M2-like macrophages had a negative association. Especially interesting was the finding that physical proximity of CD8 T cells to tumor cells also correlated with improved survival. This was not a mechanistic study, but these findings support the notion that in spite of the relatively low numbers of immune cells, adaptive anti-tumor immune responses occur in medulloblastoma and may positively impact outcome.

What was more surprising was the influence of less studied immune cells. NK cell infiltration is typically thought to be part of a “hot” tumor microenvironment. However, in this study increased NK cell density was associated with reduced overall survival. Similarly, B cell infiltration also conferred a poor prognosis. In 2 of the 249 cases evaluated B cells formed tertiary lymphoid structures within the tumors. This is the first time these structures have been described in medulloblastoma; however, low numbers preclude any conclusions about whether they are facilitating anti-tumor immune responses or not.

A number of prior studies have evaluated immune checkpoint expression on both tumor and infiltrating immune cells in medulloblastoma. In the current study two findings stand out. One is that for the first time, the authors describe TIM-3 expression by medulloblastoma tumor cells. TIM-3 expression on tumor cells is emerging as an important biomarker in a number of cancers including a recent comprehensive study in breast cancer where it was shown to facilitate metastasis through immune evasion.^4^ However, in contrast to this and other studies, in the current medulloblastoma cohort the authors found that TIM3+ tumor cells were associated with improved prognosis. This raises the question of whether TIM3 plays a fundamentally different role in medulloblastoma compared to other cancers and how this would translate clinically. Would anti-TIM3 therapies be detrimental to patients with medulloblastoma or is the presence of TIM3 on these tumor cells reflective of a “warmer” immune microenvironment? Tumoral PD-L1 was also seen in association with TIM3 and correlated with improved progression-free survival (PFS) in the cohort. It is well established that interferon gamma and other proinflammatory cytokines in hot tumor microenvironments drive PD-L1 expression on tumor cells.^5^^,^^6^ TIM3 is similarly regulated.^7^ Neither proximity of TIM3+ tumor cells to T cells or cytokine levels were specifically assessed, but interferon secretion by T cells could be a possible mechanism inferred by the other proximity data. Additional investigation into the significance and mechanism of this finding is clearly warranted as there have not yet been any preclinical or clinical studies focused specifically on TIM3 in medulloblastoma.

Finally, the association of CTLA-4 expression with poor PFS across subgroups is striking in light of the results of CheckMate 908.^8^ Across multiple pediatric brain tumors nivolumab (anti-PD-1) therapy displayed no benefit over historical controls. However, in medulloblastoma alone, the combination of nivolumab with ipilumumab (anti-CTLA-4) demonstrated a trend toward improved overall survival. These results along with that of PBTC045,^9^ which evaluated the efficacy of pembrolizumab (anti-PD-1) monotherapy in pediatric brain tumors and also showed no improvement over historical controls, have led many to conclude that immune checkpoints are ineffective in medulloblastoma. However, it is possible that CTLA-4 based therapies may be more effective in medulloblastoma and that future combination trials should consider CTLA-4 as a backbone rather than PD-1. Questions remain from the current study including the fact that despite the overall negative prognostic influence of CTLA-4 across subgroups, WNT patients had significantly more CTLA-4+ cells. Whether this finding is less influential in WNT outcomes because of increased sensitivity to chemotherapy or other factors was not able to be determined and will hopefully be clarified by future studies.

In summary, the new study by Chen et al. adds nuance to our understanding of the “cold” tumor microenvironment in medulloblastoma. Along with other recent studies it suggests that this tumor demonstrates unique immune cell infiltration features and that rather than abandoning immune based therapies in this tumor we should strive to understand the mechanisms underlying its differences to find disease-specific immunotherapy approaches that are most likely to benefit medulloblastoma patients.

## Contributors

Allison M Martin, MD wrote and approved the final manuscript.

## Declaration of interests

None.
